# The Importance of Phosphoinositide 3-Kinase in Neuroinflammation

**DOI:** 10.3390/ijms252111638

**Published:** 2024-10-30

**Authors:** Brock Wright, Samuel King, Cenk Suphioglu

**Affiliations:** 1 NeuroAllergy Research Laboratory (NARL), School of Life and Environmental Sciences, Faculty of Science, Engineering and Built Environment, Deakin University, 75 Pigdons Road, Geelong, VIC 3216, Australia; brock.wright@deakin.edu.au (B.W.); samuel.king@deakin.edu.au (S.K.); 2 Centre for Sustainable Bioproducts, School of Life and Environmental Sciences, Faculty of Science, Engineering and Built Environment, Deakin University, 75 Pigdons Road, Geelong, VIC 3216, Australia; 3 Institute for Mental and Physical Health and Clinical Translation (IMPACT), Deakin University, 75 Pigdons Road, Geelong, VIC 3216, Australia

**Keywords:** neuroinflammation, neurodegeneration, phosphoinositide 3-kinase

## Abstract

Neuroinflammation, characterised by the activation of immune cells in the central nervous system (CNS), plays a dual role in both protecting against and contributing to the progression of neurodegenerative diseases, such as Alzheimer’s disease (AD) and multiple sclerosis (MS). This review explores the role of phosphoinositide 3-kinase (PI3K), a key enzyme involved in cellular survival, proliferation, and inflammatory responses, within the context of neuroinflammation. Two PI3K isoforms of interest, PI3Kγ and PI3Kδ, are specific to the regulation of CNS cells, such as microglia, astrocytes, neurons, and oligodendrocytes, influencing pathways, such as Akt, mTOR, and NF-κB, that control cytokine production, immune cell activation, and neuroprotection. The dysregulation of PI3K signalling is implicated in chronic neuroinflammation, contributing to the exacerbation of neurodegenerative diseases. Preclinical studies show promise in targeting neuronal disorders using PI3K inhibitors, such as AS605240 (PI3Kγ) and idelalisib (PI3Kδ), which have reduced inflammation, microglial activation, and neuronal death in in vivo models of AD. However, the clinical translation of these inhibitors faces challenges, including blood–brain barrier (BBB) permeability, isoform specificity, and long-term safety concerns. This review highlights the therapeutic potential of PI3K modulation in neuroinflammatory diseases, identifying key gaps in the current research, particularly in the need for brain-penetrating and isoform-specific inhibitors. These findings underscore the importance of future research to develop targeted therapies that can effectively modulate PI3K activity and provide neuroprotection in chronic neurodegenerative disorders.

## 1. Introduction

Neuroinflammation refers to the immune response within the central nervous system (CNS) that occurs in reaction to harmful stimuli, such as infections, traumatic injury, toxins, or autoimmunity [1]. This response is characterised by the activation of resident glial cells, such as microglia, astrocytes, and oligodendrocytes, alongside nonglial cells, notably neurons, which all contribute to the neuroinflammatory cascade [2]. While the primary function of neuroinflammation is to restore CNS homeostasis and repair damage, chronic or dysregulated neuroinflammation can lead to the onset of neurodegenerative processes [3]. These processes are a consistent component in many diseases of the CNS, including Alzheimer’s disease (AD), Parkinson’s disease (PD), multiple sclerosis (MS), and amyotrophic lateral sclerosis (ALS) [4,5]. Specific causes of neurodegeneration can vary depending on the disease, with impairment mechanisms being isolated or integrated, environmental, or genetic, identified or currently inconclusive in origin, with the capability of provoking an immune response [4,5,6,7]. The production of neurotoxic protein aggregates, metabolites, and/or degeneration in such disorders is commonly associated with chronic neuroinflammation through sustained CNS immune cell activation [8].

Chronic neuroinflammation is now recognised as a core feature of neurodegenerative diseases. In conditions such as AD and PD, the persistent activation of glial cells leads to the sustained release of proinflammatory cytokines, chemokines, and reactive oxygen species (ROS), which contribute to the pathological accumulation of neurotoxic proteins, neuronal damage, and eventual cell death [3]. This prolonged inflammatory response disrupts the blood–brain barrier (BBB), recruits peripheral immune cells, and exacerbates CNS injury, resulting in phenomena such as synaptic pruning, axonal demyelination, and neuronal degeneration [8,9,10,11,12,13,14,15]. If unchecked, these processes perpetuate a cycle of damage that is central to the progression of neurodegenerative diseases.

Among the numerous molecular pathways implicated in neuroinflammation, the phosphoinositide 3-kinase (PI3K) pathway is particularly noteworthy. The PI3K family consists of three classes (Class I, II, and III): Class I PI3Ks are the most extensively studied in the context of neuroinflammation [16]. These enzymes play key roles in the regulation of core cellular functions, such as growth, metabolism, and survival by activating downstream targets Akt and mTOR [17]. The dysregulation of the PI3K pathway, particularly Class I isoforms, has been linked to various neurodegenerative diseases, where it influences not only the survival of neurons but also the activation and response of glial cells, amplifying neuroinflammatory processes [4,18].

**Figure 1:**
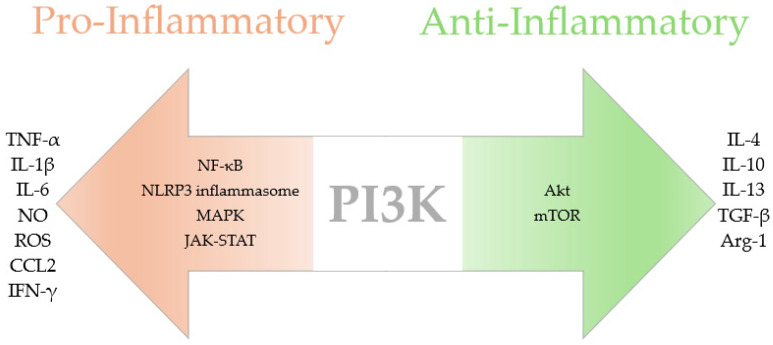
PI3K signalling in neuroinflammation.

Class I PI3K isoforms are further subdivided into four catalytic subunits (p110α, p110β, p110δ, and p110γ), each with distinct roles in the regulation of immune responses and inflammation around the body. For example, p110γ and p110δ are predominantly expressed in immune cells and are involved in controlling leukocyte recruitment and inflammatory cytokine production [19]. The PI3K signalling mechanism is placed at the centre of neuroinflammatory modulation in the CNS, balancing proinflammatory and anti-inflammatory processes (Figure 1). During proinflammation, the activation of PI3K by TLRs, cytokine receptors, and GPCRs induces the recruitment of Akt, triggering NF-κB and AP-1 transcription factors [20]. These pathways promote the release of proinflammatory cytokines, such as TNF-α and IL-1β, along with the production of chemokines, such as CCL2, that attract peripheral immune cells. Sustained NF-κB activity contributes to neuronal death and amyloid plaque accumulation and impairs tissue repair, key hallmarks of AD and MS [21]. Additionally, PI3K-mediated Akt signalling influences ROS production via iNOS, inducing oxidative stress and further amplifying neuroinflammation [22].

Conversely, anti-inflammatory signalling via PI3K/Akt/mTOR activation promotes the release of IL-10 and TGF-β to dampen proinflammatory responses and promote tissue repair [23]. Akt also inhibits NF-κB through the phosphorylation of IκB, preventing excessive inflammation. Through anti-inflammatory signalling, PI3K is known to modulate proinflammation, preventing long-term damage and promoting neuroprotection. The PI3K signalling pathways crosstalk with AP-1, NF-κB, mTOR, and IRF7, enabling a dynamic switch between immune activation and resolution, and ensuring that inflammatory responses are terminated when no longer needed [24]. This dual role of PI3K driving and resolving neuroinflammation makes it a crucial target for therapeutic intervention.

Although prevalent worldwide, there is currently no cure for early or advanced stages of neurodegenerative disorders. Research into neurodegeneration and the functionality of neuroinflammatory activation pathways and perspective dysfunction has indicated that PI3K’s involvement in neuroinflammatory mechanisms may act as the fundamental component prompting the onset and exacerbation of neurodegenerative disorders [4,25,26,27].

This review aims to provide a comprehensive overview of how PI3K signalling pathways influence neuroinflammatory processes across different CNS cell types, including microglia, astrocytes, oligodendrocytes, and neurons. By examining the roles of the various PI3K isoforms in both protective and pathological responses, we aim to clarify their contributions to neurodegenerative diseases and explore potential therapeutic strategies that target PI3K dysfunction. Understanding the role of PI3K in regulating neuroinflammation offers valuable insights into its potential as a therapeutic target, amid the expanding interest in PI3K inhibitors for addressing neurodegenerative diseases.

## 2. Phosphoinositide 3-Kinase

Phosphoinositide 3-kinase (PI3K) is a family of cytoplasmic lipid kinases important in a range of cellular functions, including signal transduction, intracellular vesicle trafficking, and cytoskeleton reorganisation. PI3K signalling is initiated by extracellular signals, such as cytokines, growth factors, hormones, and lipopolysaccharides, which activate the enzyme, leading to a cascade of downstream signalling events that regulate cell survival, proliferation, and immune responses [4,18,28,29]. PI3K is essential for maintaining cellular homeostasis and immune functions, particularly in the context of neuroinflammation, where it has a dual role in both promoting neuroprotection and exacerbating neurodegeneration when dysregulated [30].

**Figure 2:**
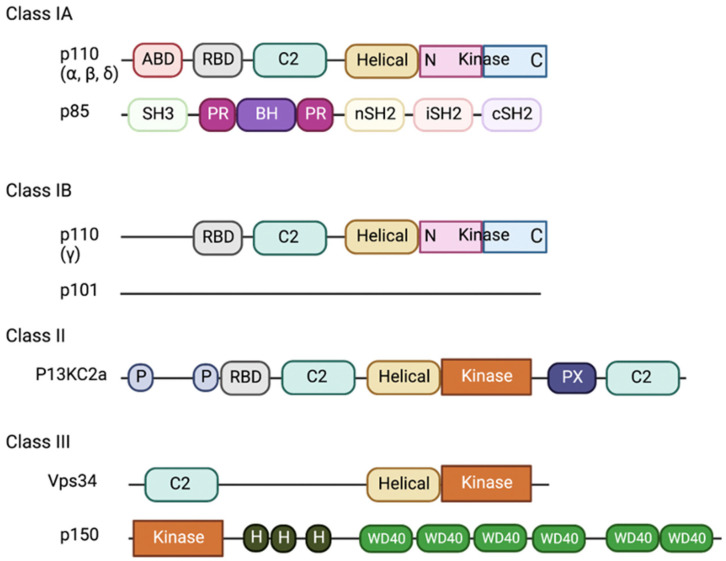
Structural illustration of catalytic and regulatory subunits of PI3K Class I, II, and III. All PI3K catalytic subunits comprise a two-lobe (N and C) kinase, a helical domain, a lipid-binding C2 domain, and a Ras-binding domain (RBD). Class IA catalytic isoforms display an adaptor binding domain (ABD). Regulatory p85 group adaptors contain a SH3 domain at the N-terminal. Class IB PI3K isoforms possess adaptors of p101, sharing regions of low homology (H1 and H2). Class II PI3K exhibit extended N-terminal portions containing proline-rich motifs (P) followed by RBD, C2, and helical domains, along with Phox homology (PX) and an extra C2 domain. Class III contains C2 and helical domains; however, they lack RBD, instead binding to corresponding the p150 adaptor containing a kinase domain followed by heat (H) and aspartic/tryptofan40 (WD40) domains. Adapted from [35].

The PI3K family is divided into three classes (I, II, and III) based on structural differences and substrate specificity, where Class I PI3Ks are the most well-studied in relation to inflammation and neurodegeneration [31,32]. The role of Class I PI3Ks is to convert phosphatidylinositol-4,5-bisphosphate (PI(4,5)P2) into phosphatidylinositol-3,4,5-trisphosphate (PI(3,4,5)P3), a critical second messenger that activates various downstream signalling pathways [31]. Class I PI3Ks are further divided into two subfamilies based on catalytic and regulatory subunits, Class IA (PI3Kα, PI3Kβ, PI3Kδ) and Class IB (PI3Kγ) (Figure 2) [28,33,34].

Each Class I PI3K isoform is extremely important due to its association with various cellular functions extending into all cell types and developmental stages [32]. PI3Kα and PI3Kβ are expressed ubiquitously and are involved in regulating cellular metabolism and growth through p21 and p27 signalling pathway activation, additionally inactivating p53 and Bcl-xL evading apoptosis, thus PI3Kα and PI3Kβ are frequently implicated in cancer and cell proliferation [34]. PI3Kδ and PI3Kγ are primarily expressed in immune cells and play crucial roles in regulating immune cell activation and inflammation. PI3Kδ is central to adaptive immune responses (T- and B-cell function), while PI3Kγ regulates the inflammatory response in myeloid cells, such as macrophages and microglia [36,37].

### 2.1. Signalling Pathways of PI3K

Once activated, PI3K generates PI(3,4,5)P3, which serves as a docking site for downstream kinases, including 3-phosphoinositide-dependent protein kinase-1 (PDK1) and Akt (protein kinase B) [38]. The activation of Akt is a critical event in the PI3K pathway, leading to the phosphorylation of multiple substrates involved in cell survival, growth, metabolism, and motility [18,30,34]. The PI3K/Akt pathway mediates cell survival by phosphorylating proapoptotic proteins, such as BAD, caspase-9, and forkhead transcription factors, and promoting the expression of antiapoptotic factors, such as Bcl-2 [39,40]. Cell growth and proliferation is achieved through the activation of PI3K/mTORC2 (mammalian target of rapamycin), while PI3K/AKT/mTORC1, or PAM, regulates protein synthesis and cell growth by controlling ribosomal biogenesis and translation [41]. During inflammation, Akt signalling inhibits the NF-*κ*B pathway, a critical regulator of inflammatory responses, thereby controlling the production of proinflammatory cytokines [42,43]. However, dysregulation of this pathway can contribute to chronic neuroinflammation and neurodegeneration conditions.

Akt, a pivotal downstream effector, engages in phosphorylation cascades affecting various targets, including BAD, IKK, Mdm2, and FOXOs. PI3K/Akt induces GSK3 inhibition, advancing cell proliferation and iNOS, as well as PI3K/Akt activating IKK, thus regulating angiogenesis via nitric oxide (NO) production involved in cancer pathogenesis [18]. Connections with multiple pathways, such as chemokine, focal adhesion, and TLR receptor signalling, highlight PI3K pathway versatility [30]. PI3K/Akt activation influences various processes: mTOR and p70S6Kinase in protein synthesis, NF-*κ*B in inflammatory response, BAX and caspase-3 in apoptosis, CREB and BCL2 in cell survival, GSK3 in glycogen synthesis, FOXO in cell cycle, and PRAS40 in cell growth [23,31,32,33], emphasising its central role in diverse cellular outcomes.

### 2.2. PI3K Involvement in Disease

PI3K Class I isoforms have a significant association with a range of human disorders with characteristics of either augmented or decreased levels of PI3K activity (Table 1) [34].

The PI3K⍺ isoform is distinct in cellular processes, regulating normal growth and developmental processes. Gain-of-function mutations or the overactivation of PI3Kα, therefore, drives oncogenesis in cancers, such as breast and lung cancer. Somatic mutations in PI3Kα are also central to PROS (PIK3CA-related overgrowth syndrome) and CLOVES (congenital lipomatous overgrowth, vascular malformations, and epidermal nevi) disorders causing hyperactive PI3K signalling leading to tissue overgrowth, disfigurement and organ dysfunction [44,45]. PI3K⍺ plays a prominent role in oncogenesis, driving cell proliferation and survival; therefore, PI3K inhibitors targeting the α-isoform are being developed for cancer therapies.

PI3Kδ and PI3Kγ isoforms are essential in regulating immune responses. PI3Kδ is mainly expressed in B-cells and T-cells and is linked to immune deficiencies and autoimmune disorders. Mutations and hyperactivity of PI3Kδ lead to immune regulatory disorders, such as activated PI3Kδ syndrome (APDS), where patients experience recurrent infections and autoimmunity due to improper immune signalling [33,46]. Additionally, PI3Kγ is evident in myeloid cells, playing a role in immune cell recruitment and antibody production. Loss-of-function mutations of PI3Kγ cause impaired macrophage and neutrophil function as well as immune cell recruitment and antibody production, reducing the body’s ability to mount an effective immune response [47].

PI3Kβ has been observed in combination with other PI3K isoforms; however, there is limited knowledge about this isoform’s disease involvement. PI3Kβ is involved in tumour cell survival through overactivation linked to cancer, including prostate and breast cancer. PI3Kβ activation mutations promote metastasis and tumour resistance by enhancing PI3K/Akt signalling.

In AD and PD, aberrant PI3K signalling plays a protective role; however overprotection may contribute to neuronal damage and chronic inflammation. In AD and PD, environmental stimuli, such as amyloid-β plaques and oxidative stress, trigger PI3K/Akt/NF-κB signalling, promoting microglia and astrocyte activation. The overactive dysfunction of this response can increase neuroinflammatory spread, damage neurons, impair Aβ clearance, as well as contribute to α-synuclein accumulation [48]. On the other hand, the underactivation of PI3K/Akt, often due to PTEN hyperactivity, reduces mTOR signalling and Bcl-2 expression, impairing cell survival and the clearance of aggregates.

**Table 1:** Class I PI3K isoforms and human disease involvement.

PI3K Isoform	Disease Involvement	References
PI3K	Hematopoietic cancers and neurological disorders	[49,50]
Alpha (p85)	SHORT syndrome	[51]
Alpha (PI3Kα)	Colorectal cancer	[52,53]
	Breast, glioblastoma, gastric, and lung cancers	[52,54]
	Breast and ovarian cancers	[55,56,57]
	Cancers	[58]
	Hyperglycaemia	[59]
	CLOVES syndrome	[44]
	PROS disorder and polycystic kidney disease	[45,60,61]
Delta (PI3Kδ)	Chronic lymphocytic leukemia and non-Hodgkin lymphoma	[62,63]
	Breast cancer and B-cell malignancies	[64]
	APDS disease and PASLI disease	[65,66,67]
	Autoimmune diseases	[46]
Gamma (PI3Kγ)	Rheumatoid disease, multiple sclerosis, COPD, asthma, allergic disease, hypertension, systemic lupus erythematosus, atherosclerosis, cardiac failure, pancreatitis, thrombosis, and Stroke	[68,69,70]
	Inactivated PI3K-γ syndrome (IPGS)	[47]
	Cardiovascular disease and stroke (thrombotic disease, MI, atherosclerosis)	[71]
	Inflammatory diseases and allergies (acute pancreatitis, allergy anaphylaxis, asthma, COPD)	
	Autoimmune diseases (rheumatoid arthritis, SLE, multiple sclerosis, EAE)	
	Cancers (solid, haematological)	
Alpha/Beta/Gamma	Diabetes	[72]
Alpha/Gamma	Cardiovascular disease	[73]
Delta/Gamma	Asthma, rheumatoid arthritis, and cancers	[74,75]

### 2.3. PI3K Signalling During Neuroinflammation

In neurodegenerative diseases, such as MS, AD, and PD, PI3K signalling plays a complex role in regulating both protective and detrimental responses within the CNS. PI3K/Akt signalling can be neuroprotective by promoting neuronal survival, glial cell support, and limiting excessive inflammation [76,77]. However, the dysregulation of PI3K signalling, particularly in microglia and astrocytes, can exacerbate neuroinflammation and contribute to neurodegeneration.

PI3K acts as a key regulator for mechanisms associated with inflammatory responses stimulated by extracellular prompts, thus illustrating PI3K as a potential target to mitigate the progression of unwanted proinflammatory neuroinflammation [3,62]. The PI3K signalling network is evident in a range of immune responses throughout the body, and further investigation indicates that PI3Kδ and PI3Kγ isoforms are enriched in immune cells, such as microglia [62,63].

PI3Kγ is critical in microglial activation, modulating microglial migration, cytokine production, and phagocytic activity. During neuroinflammation, PI3K signalling can suppress excessive proinflammatory responses through Akt/NF-κB inhibition. Still, overactivation can lead to sustained microglial activation, contributing to the progression of diseases such as MS. PI3Kδ regulates astrocyte responses during inflammation, including the formation of glial scars and the modulation of the BBB. Dysregulated PI3K in astrocytes impairs barrier function, allowing peripheral immune cells to enter the CNS and exacerbating neuroinflammation. PI3K/Akt signalling in neurons is crucial for synaptic plasticity and survival. In diseases such as AD, PI3K dysregulation contributes to synaptic dysfunction and neuronal death. Additionally, the chronic activation of PI3K/Akt in neurons can lead to oxidative stress and mitochondrial dysfunction, further driving neurodegeneration.

The PI3K signalling pathway is regulated by positive and negative feedback loops, balancing immune responses to maintain homeostasis. The positive feedback loop of PI3K/Akt activation induces the NF-κB pathway, promoting proinflammatory cytokine release. The chronic stimulation of TLRs and cytokine receptors can also induce persistent PI3K/Akt/NF-κB activation, creating feedback loops that amplify the release of proinflammatory cytokines, impairing immune resolution and promoting immune cell overactivation.

Additionally, damage-associated molecular patterns (DAMPs) released from damaged neurons or glial cells bind to TLR4 receptors, escalating the inflammatory response through the PI3K-mediated activation of NF-κB and furthering cytokine release [78]. These feedback mechanisms spread neuroinflammation by keeping glial cells in a proinflammatory state, releasing ROS and cytokines. It has been shown that amyloid plaques can disrupt PI3K signalling by stimulating both NF-κB and mTOR, enhancing glial activation and preventing the switch to an anti-inflammatory state, highlighting PI3K’s association with AD progression [79].

On the other hand, PI3K/Akt signalling also prompts negative feedback loops to limit excessive neuroinflammation. In an anti-inflammatory context, Akt inhibits NF-κB through IκB, preventing the downstream translocation of proinflammatory cytokines. PI3K/Akt activation stimulates mTOR, promoting inflammation resolution by shifting immune cells to an anti-inflammatory state indicated by the expression of Arg-1 and IL-10 [43]. Furthermore, IL-10 enhances anti-inflammatory signalling by activating Akt to dampen NF-κB activity and drive the expression of repair-associated molecules [80].

Phosphatase and tensin homology (PTEN) normally acts as a negative regulator of PI3K by dephosphorylating PIP3, ensuring the controlled activation of Akt/mTOR and Akt/NF-κB pathways to promote anti-inflammatory responses. However, PTEN loss-of-function mutations or inhibition by ROS results in uncontrolled PI3K-Akt activation, leading to excessive proinflammatory signalling and oxidative stress [81].

Aging has also been shown to impact the functionality of PI3K. Increased activity in PTEN excessively suppresses PI3K-Akt-mTOC, limiting the production of anti-inflammatory mediators and impairing cellular repair [82]. Simultaneously, NF-κB hyperactivation in aged glial cells fuels chronic neuroinflammation, worsening neuronal damage [83]. Additionally, the accumulation of senescent cells exacerbates this imbalance between inflammatory responses, inducing proinflammatory cytokine and chemokine release, thus further disrupting PI3K’s regulatory function, preventing immune response modulation, and in turn, driving neuroinflammation and neurodegeneration [84].

## 3. Neuroinflammatory Cell Types

Neuroinflammation is mediated by the production of cytokines, chemokines, ROS, and secondary messengers. Such mediators are produced and released by resident CNS glia notably microglia, astrocytes and oligodendrocytes, and neuronal cells as well as peripherally derived immune cells. The involvement of these cells formulates the intricate mechanism of neuroinflammation.

The interactions between these CNS cell types during neuroinflammation are complex and highly coordinated. Microglia–astrocyte crosstalk is a prime example of a feedback loop in which PI3K signalling can modulate inflammatory outcomes. Activated microglia release cytokines, such as TNF-α and IL-1β, which induce A1 astrocytes, leading to neurotoxicity [48,85]. Conversely, PI3K-mediated anti-inflammatory signals in microglia can induce A2 astrocytes, which promote neuroprotection and inflammation resolution [85]. The oligodendrocyte–microglia interaction is another critical feedback mechanism in MS, where PI3K signalling helps regulate the immune environment that either fosters or impairs remyelination [86,87].

### 3.1. Microglia

Microglia are the brain’s primary immune cells, tasked with maintaining CNS homeostasis and responding to injury or infection. As resident macrophages, they continually monitor the microenvironment, and upon detecting harmful stimuli, they shift to an activated state, adopting ameboid morphology and upregulating receptors associated with the innate immune response [88,89]. During this activation, microglia migrate to injury sites and phagocytose cellular debris, abnormal protein aggregates and release proinflammatory cytokines (e.g., TNF-α, IL-1β) and chemokines, initiating a cascade of immune responses [89]. Microglial cells exhibit a high degree of heterogeneity and plasticity, heavily influenced by the microenvironment during neuroinflammation and CNS recovery [90,91].

**Figure 3:**
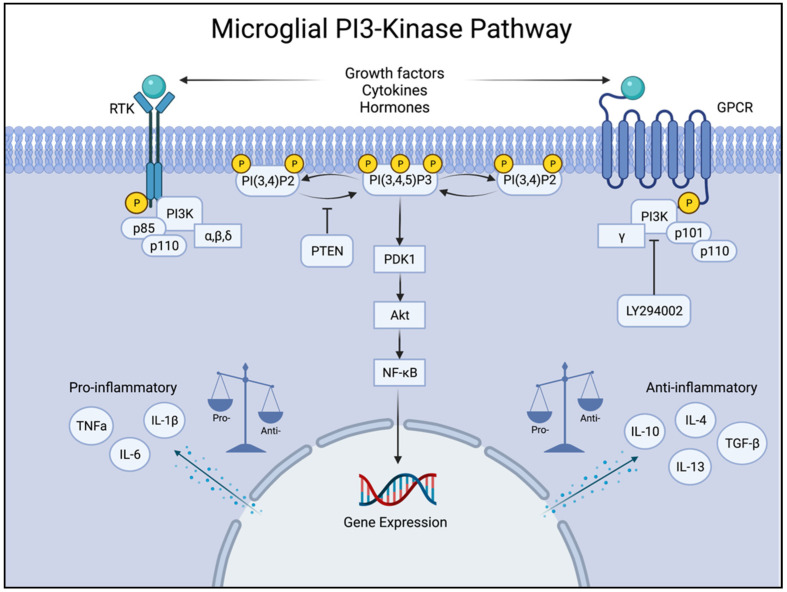
The microglial PI3K pathway. RTK and GPCR activation from extracellular signals, growth factors, cytokines, and hormones, inducing tyrosine kinase domains aiding in the recruitment and activation of Class I PI3K. Phosphorylation of PI(4,5)P2 to PI(3,4,5)P3 leads to the activation of PDK1, Akt, and NF-*κ*B. The activation of NF-*κ*B induces gene expression of either pro- or anti-inflammatory mediators, potentially leading to an imbalance in proinflammatory mediators.

The PI3K pathway is crucial in regulating microglial activation and inflammatory responses (Figure 3). Particularly, PI3Kγ modulates microglial migration, cytokine production, and phagocytic activity.

Additionally, PI3K signalling plays a key role in mediating the M1/M2 polarisation of microglia [92,93]. The main basis for distinguishing between M1 and M2 microglia lies in their biological functions and secreted cytokines and chemokines [94]. In M1 microglial polarization, proinflammatory phenotype, imbalanced Akt activity leads to the activation of NF-κB and MAPK pathways, driving the release of cytokines, such as TNF-α, IL-1β, and iNOS, along with CCL2, which attract peripheral immune cells and sustain neuroinflammation [95,96]. PI3K also influences the activity of GSK-3β, a kinase that drives M1 responses when active; however, inhibition by Akt shifts microglia toward M2 by enhancing anti-inflammatory signalling [97].

In contrast, M2 microglia adopt an anti-inflammatory phenotype, by which PI3K/Akt activation inhibits NF-κB through phosphorylating IκB, preventing nuclear translocation, and activating the mTOR pathway, which promotes anti-inflammatory responses [98]. PI3K/Akt also upregulates arginase-1 (Arg-1), TGF-β, IL-10, and CD206, key markers of M2 microglia, which facilitate inflammation resolution and promote tissue repair [95,96]

These regulatory dynamics highlight the central role of PI3K in modulating microglial functions and suggest its potential as a therapeutic target for controlling microglial overactivation in neurodegenerative diseases [1,99,100].

### 3.2. Astrocytes

Astrocytes are the most abundant glial cells in the CNS and are essential for maintaining brain homeostasis. Astrocytes play a key role in maintaining normal CNS health and function by achieving diverse functions, including neurotransmitter recycling, BBB maintenance, metabolic support, and synaptic regulation [101,102]. Astrocytes aid in the formation of the BBB in addition to adjusting blood flow, ATP, and glucose supplies, ensuring normalised brain function [103]. During neuroinflammation, astrocytes undergo reactive gliosis, characterised by hypertrophy, the upregulation of intermediate filament proteins (e.g., GFAP), and the release of inflammatory cytokines [101,103]. The ablation of astrocytes following CNS injury indicates sustained inflammation, impaired BBB repair, and increased neurodegeneration [81].

PI3K signalling regulates astrocyte activation, influencing their role in neuroinflammation. PI3K/Akt pathways are involved in astrocyte survival, activation, and cytokine release. In the context of inflammatory CNS diseases, astrocytes use PI3K signalling to reinforce the BBB and regulate the entry of peripheral immune cells into the CNS [104]. Astrocytes’ involvement in BBB integrity is critical, as they help control the influx of immune cells during neuroinflammation, which can either mitigate or exacerbate neuronal damage.

The PI3K signalling pathway is critical for influencing astrocyte polarization by which the A1 phenotype is proinflammatory and the A2 phenotype is anti-inflammatory [101,105,106,107]. PI3K/Akt signalling activates NF-κB and STAT3, which promote the release of TNF-α, IL-1β, and C1q. A1 activation can additionally be induced by microglial-released cytokines, promoting an increased proinflammatory profile [108].

PI3K/Akt/mTOR signalling favours the A2 phenotype, promoting the production of IL-10, TGF-β, and neurotropic factors, such as BDNF, enhancing neuronal survival and repair, exhibiting neuroprotection, reinforcing the BBB, and deterring peripheral immune cell infiltration [105,107,109,110]. Driving the anti-inflammatory A2 phenotype, Akt inhibits GSK-3β, downregulating NF-κB activity and activating mTOR [97]. Furthermore, STAT3 activation, when paired with mTOR signalling, facilitates the switch to A2 by promoting astrocytic support of neuronal regeneration. Crosstalk between Akt/mTOR and JAK/STAT pathways fine-tune the polarization of astrocytes, with the inhibitory regulation of NF-κB preventing excessive A1 activation [105].

During chronic inflammation or injury, astrocytes aid in scar formation to isolate damaged areas from healthy tissue in turn, promoting tissue repair [111,112]. However, excessive scarring may impede axonal regeneration [113]. Moreover, astrocyte–neuron communication during neuroinflammation is partly regulated by PI3K signalling. Astrocytes interact with neurons during inflammation, influencing their function and modulating synaptic transmission. The intricate and diverse functions of astrocytes highlight their essential role in both normal CNS function and neuroinflammatory conditions, emphasising their significance in maintaining CNS health.

### 3.3. Oligodendrocytes

Oligodendrocytes are glial cells essential for myelination in the CNS, facilitating electric potential propagation and providing metabolic support to neurons [114]. Apart from their primary role in myelinating axons, oligodendrocytes are involved in the neuroinflammatory response and express receptors for immunomodulatory molecules, allowing them to sense inflammation. Oligodendrocytes also attract immune cells via IL-8 and CCL2 during acute inflammation. Thus, associated with microglial recruitment to damage tissues, oligodendrocytes interact with immune cells during neuroinflammation and participate in immune signalling pathways.

During neuroinflammation, oligodendrocytes are vulnerable to damage and degeneration. The release of proinflammatory cytokines and ROS can directly or indirectly lead to oligodendrocyte death or dysfunction, resulting in demyelination [87,93]. PI3K signalling is critical for oligodendrocyte survival and myelination. The PAM pathway regulates oligodendrocyte differentiation and the synthesis of myelin. In MS, impaired PI3K signalling can hinder the repair of damaged myelin and the differentiation of oligodendrocyte precursor cells (OPCs) into mature myelinating cells [115,116]. This failure to remyelinate damaged axons contributes to persistent neurological deficits and disease progression. PI3K signalling also mediates oligodendrocyte protection during inflammation by promoting the expression of antiapoptotic factors, ensuring that oligodendrocytes can survive a proinflammatory environment and participate in the repair processes [117]. Oligodendrocytes also play a role in neuroprotection by releasing neurotrophic factors supporting the survival and health of nearby neurons. Such dysfunction or loss of these protective mechanisms during neuroinflammation can contribute to secondary neuronal damage. Furthermore, in response to neuroinflammation and demyelination, astrocytes and oligodendrocytes may undergo reactive gliosis and form glial scars, containing localised damage at the cost of impeding axonal regeneration [118].

### 3.4. Neurons

Neurons are the core functional units of the CNS, responsible for transmitting electrical and chemical signals throughout the brain and body. However, neurological disorders can arise when neurons malfunction, become damaged, or degenerate, emphasising their crucial role in the health and function of the body.

While not traditionally involved in immune responses, neurons can become active participants in neuroinflammation by expressing cytokine and chemokine receptors and releasing inflammatory mediators, such as HMGB1 [119,120]. Neurons possess functional TLRs, which can contribute to the immune response once activated, producing cytokines and chemokines [121]. In response, neurons can synthesise and express TNF-α and IL-1β, playing a role in neuronal communication between glial and nonglial cells [122]. Such inflammatory responses can lead to excitotoxicity, where the excessive release of glutamate induces the overstimulation of neurons, resulting in neuronal damage or death [123,124].

PI3K/Akt signalling plays a neuroprotective role by promoting neuronal survival, growth, and synaptic plasticity [125]. The intricate interplay between neurons and the immune response underscores the complex nature of neuroinflammation. The role of neurons as both recipients and responders to inflammatory signals highlights their importance in the CNS and emphasises the need for research into strategies that can preserve neuronal health and function in the face of neuroinflammatory challenges.

## 4. PI3K in CNS Immune Cells

PI3K is central to the regulation of CNS immune cells, playing a critical role in cell survival, differentiation, and inflammatory responses. The dysregulation of PI3K signalling in microglia, astrocytes, oligodendrocytes, and neurons can drive chronic neuroinflammation and contribute to neurodegenerative disease pathology, including AD, PD, MS, and ALS. Targeting PI3Kγ and PI3Kδ isoforms offers a promising approach for selectively modulating neuroinflammation and minimising off-target effects.

The activation of various Class I PI3K isoforms yields contrasting effects on proinflammatory cytokine production. TLR-induced recruitment of Class I PI3K has been found to suppress NF-*κ*B-driven proinflammatory cytokine production in macrophages. However contrasting studies reveal that PI3K deficiency in dendritic cells enhances proinflammatory cytokine secretion and diminishes anti-inflammatory cytokine production [59]. p38 MAPK governs cytokine and proinflammatory mediator production, alongside iNOS and COX-2 expression in LPS-induced microglia. Additionally, environmental stressors and inflammatory cytokines activate SAPK/JNK inflammation [59]. Importantly, the PI3K/Akt pathway negatively regulates NF-*κ*B activation and inflammatory cytokine expression, potentially impacting neuroinflammation and CNS disease neuropathology [60].

The association between neuroinflammation and PI3K/Akt presents a significant importance in determining the pathogenesis of chronic neuroinflammation. Dysregulation of the PI3K/Akt pathway in neuronal cells may lead to detrimental effects including membrane depolarisation, increased ROS levels, mitochondrial fragmentation, and oxidative phosphorylation. Most neurodegenerative disorders comprise pathological elements that possess the ability to stimulate TLR4 and sustain PI3K/Akt activation within glial cells, therefore, inducing the NF-*κ*B-dependent transcription of proinflammatory mediators. The association of the PI3K/Akt pathway with neuroinflammation paired with the availability of multiple stimuli to activate the pathway alludes to PI3K being a key molecule in chronic neuroinflammation with relation to the development and progression of neuronal damage [63].

### 4.1. Microglia and PI3K Signalling

PI3K signalling is crucial for regulating microglial activation, polarisation, and phagocytosis. The PI3Kδ isoform, predominantly expressed in microglia, is critical for the M1/M2 polarisation of these cells [4]. M1 microglia exhibit a proinflammatory phenotype, releasing cytokines, such as TNF-α and IL-1β, while M2 microglia adopt an anti-inflammatory phenotype, promoting tissue repair through factors, such as IL-10 [30,126,127,128]. The dysfunction of the PI3K/Akt pathway can drive the M1 microglial phenotype, contributing to elevated proinflammatory cytokines associated with prompting a positive feedback loop causing chronic neuroinflammation [4,30]. Additionally, the dysregulation of PI3Kδ signalling can exacerbate neuroinflammatory conditions, such as MS, where sustained activation of M1 microglia contributes to demyelination and axonal damage.

PI3K stimulation in microglial cells, inducing neuroinflammation, can result from several key environmental stimuli. Pathogen-associated molecular patterns (PAMPs), such as lipopolysaccharides (LPS) and viral RNA/DNA, activate TLRs triggering PI3K-Akt/NF-κB-mediated proinflammatory cytokine production [78]. Similarly, DAMPs, including amyloid-β plaques, ATP, and mitochondrial DNA released from injured cells, engage receptors that activate the PI3K pathway [129]. Chronic exposure to proinflammatory cytokines also sustains PI3K signalling through receptor engagement, driving microglial activation and perpetuating neuroinflammation [90]. These stimuli overwhelm microglial homeostasis, maintaining a proinflammatory M1 state, which ultimately contributes to the progression of neurodegeneration.

PI3Kγ regulates microglial migration and recruitment to injury sites by expressing the complement component C5a; as well as microglial proliferation as seen in PI3Kγ knockout and knockdown mice, microglia had a significant impairment to proliferation and reduced viability [37,130]. The overactivation of PI3Kγ through stimuli can lead to excessive microglial infiltration exacerbating neuroinflammation; therefore, inhibiting this isoform may limit immune cell infiltration into the CNS [27,37,131].

### 4.2. Astrocytes and PI3K Signalling

Astrocytes maintain CNS homeostasis and respond to injury by becoming reactive, a process that can either be protective or detrimental, depending on the context. PI3K signalling regulates astrocytic responses during neuroinflammation, influencing cytokine production, glial scarring, and the integrity of the BBB [132,133,134].

PI3Kδ and PI3Kγ are involved in regulating the production of proinflammatory mediators in astrocytes. The activation of the PAM pathway induces the production of IL-6, IL-1β, and TNF-α, which amplifies neuroinflammation in diseases such as AD and PD [134,135]. Additionally, PI3K activation triggers astrocyte-mediated glial scar formation in response to CNS injury, which can be beneficial by containing inflammation but can also impede axon regeneration in chronic conditions such as MS. Experimental autoimmune encephalomyelitis (EAE) models illustrate the astrocyte upregulation of transcription factors for inflammatory mediators, notably C-C motif chemokine ligand 2 (CCL2), CCL5, C-X-C motif chemokine ligand 10 (CXCL10), iNOS, and TNF, but conversely, reducing anti-inflammatory cytokines IL-10 and IL-27 when compared to control mice [134]. The EAE model demonstrates the involvement of PI3K in the release mechanisms of astrocytes showing that CCL2, CXCL10, and vascular endothelial growth factor (VEGF) are regulated by NF-*κ*B, while BDNF (brain-derived neurotrophic factor) enhances STAT3 activation [134]. In addition, the neuroprotective role of astrocytes can be shown by BDNF production, alleviating axonal damage in EAE models [134].

PI3K signalling mediates the interaction between astrocytes and microglia. Astrocyte-derived cytokines can modulate microglial activity, either promoting or dampening inflammation depending on the signalling context. For example, astrocyte-derived BDNF can promote M2 microglial polarisation, reducing neuroinflammation and supporting neuronal repair [134]. Proinflammatory signals activate PI3K, leading to the promotion of A1-specific genes, such as complement component 3 (C3) and other neurotoxic factors, contributing to an inflammatory environment causing neuronal damage and dysfunction. Conversely, suppressing PI3K induces the promotion of the A2 phenotype in response to anti-inflammatory signals, thus expressing A2-specific genes, such as Arg1 and chitinase-3-like protein (Ym1), in addition to other anti-inflammatory and neuroprotective factors [107,108].

Isoform-specific inhibitors targeting PI3Kδ and PI3Kγ in astrocytes could reduce proinflammatory cytokine production and glial scarring, potentially benefiting patients with AD, PD, and MS by preventing further neuronal damage, while promoting repair processes.

### 4.3. Oligodendrocytes and PI3K Signalling

Oligodendrocytes are responsible for the myelination of CNS axons, which is critical for efficient signal transmission. PI3K signalling plays a key role in the differentiation, survival, and function of oligodendrocytes, especially in neuroinflammatory conditions such as MS, where demyelination is a hallmark. The PI3K/Akt pathway activation is crucial for oligodendrocyte survival and axonal myelination, as demonstrated in EAE models [99,136,137]. PI3K signalling activation can influence the viability of oligodendrocyte, as inflammatory mediators may impact survival. PI3K/Akt pathway involvement in oligodendrocyte differentiation and myelination suggests that, during neuroinflammation, PI3K signalling may affect the maturation of OPCs and their ability to myelinate axons [136,138]. Therefore, the dysregulation of this pathway impairs the remyelination process in diseases such as MS, contributing to prolonged demyelination and neurological dysfunction [139]. Inflammatory cytokines, such as TNF-α, can inhibit OPC differentiation, and IL-1, -6, -12, -23, and IFNγ can exacerbate neuronal damage due to elevated expression in AD and MS [140,141].

Oligodendrocytes, often viewed as targets of detrimental inflammation, are also key immune cells within the CNS, having been shown to express receptors for proinflammatory cytokines. As a result of oligodendrocyte degradation caused by cuprizone-induced demyelination, TLR3 activation leads to OPCs upregulating IL-1β, CCL2, and CXCL10 [142], playing a role in regulating oligodendrocyte function and survival. Such proinflammatory mediators act to stimulate OPC migration and attract microglia cells as well as upregulation being observed in the models of white matter abnormalities [142].

### 4.4. Neurons and PI3K Signalling

Neurons are central to all CNS activities, and their health is critically dependent on balanced signalling pathways. PI3K activation in neurons promotes survival, synaptic plasticity, and neurotransmitter release. The PI3K/Akt/mTOR signalling pathway influences synaptic vesicle recycling by regulating GTPases and proteins involved in vesicle fusion, promoting neurotransmitter release [143]. PI3K/Akt signalling modulates the activity of synapsin, which tethers vesicles to the cytoskeleton, aiding in mobilisation during synaptic transmission [144]. Additionally, PI3K/mTOR signalling controls the trafficking of the AMPA receptor involved in receptor insertion and retention at the synapse [145].

As a result, disruptions in the PI3K signalling pathways have been linked to increased neuronal vulnerability to apoptosis and synaptic dysfunction. PI3K also modulates neuronal responses to inflammation by regulating the production of antiapoptotic proteins, such as Bcl-2, and ensuring the maintenance of synaptic integrity in the face of inflammatory stress.

PI3Kα is important for neuronal survival and the regulation of synaptic plasticity. The activation of PI3K/Akt in neurons leads to the phosphorylation of downstream targets, such as mTOR, which supports cell survival by promoting protein synthesis and preventing apoptosis [48,146]. PI3Kγ, on the other hand, has a role in modulating the neuronal response to chemotactic and migrating cues during development or in response to certain inflammatory signals [48].

Inflammatory processes can induce the activation of PI3K in neurons through the expression of proinflammatory receptors. Consequently, PI3K activation can impact neuron excitability and synaptic transmission, leading to changes in neuronal firing patterns and synaptic plasticity during neuroinflammation [147]. In diseases such as AD and PD, the overactivation or dysregulation of the PI3K pathway can contribute to synaptic dysfunction and neuronal death by impairing mitochondrial function and increasing oxidative stress.

### 4.5. Dysfunction of PI3K Signalling

Dysregulated PI3K signalling contributes significantly to the progression of neurodegenerative diseases. Chronic neuroinflammation, driven by sustained PI3K/Akt activation, creates a feedback loop where neuronal damage triggers the further activation of immune cells, perpetuating neuroinflammation and leading to synaptic loss, demyelination, and neuronal death.

Neuroimmunomodulation highlights the mechanism that damaged signals induce neuroinflammatory responses transcribing NF-*κ*B. Subsequently aiding the overproduction of proinflammatory cytokines as well as additional activation of glial and neuronal receptors. Further evidence of this mechanism indicates that damaged neurons can re-activate the CNS immune response, inducing a positive feedback cascade of continuous proneuroinflammation [113]. Severe neuroinflammation prompted by external factors, dysfunction, and mutations can subsequently instigate damage to neuronal tissue linked to the development and progression of various neurodegenerative diseases [16,17,28].

PI3K/Akt signalling is recognised as a key modulator of microglial activity, illustrating that dysfunction to this pathway in microglia skews the balance toward a prolonged M1 proinflammatory state, contributing to neurotoxicity [30,148]. In astrocytes, PI3K activity promotes glial scarring through astrocyte migration and can impair BBB integrity through the PI3K/Akt/eNOS, producing excessive NO, leading to further neuronal injury [149,150]. Impaired PI3K signalling disrupts the ability of OPCs to differentiate and remyelinate axons, worsening outcomes in MS [149,151]. Dysregulated PI3K/Akt signalling in neurons can increase susceptibility to apoptosis and oxidative stress, key contributors to AD and PD progression [18,48].

Considering the central role of PI3K in regulating inflammatory responses in astrocytes, microglia, and oligodendrocytes, PI3K has been considered a potential therapeutic target for modulating immune responses during neuroinflammatory disorders (Table 2). Investigating the interactions between PI3K and downstream signalling pathways, such as NF-*κ*B, MAPK, STAT, and mTOR, is necessary to fine-tune the behaviours and immune responses of these cell types.

## 5. Regulation of PI3K

Ineffective neuroinflammation causes significant impact due to the CNS being the control centre of the body. It is important to establish the process of neuroinflammation, and when neuroprotection becomes counterproductive, by manipulating PI3K activity and its regulation, this can assist in negating or limiting irreversible damage to the CNS.

PTEN facilitates the downregulation of the PI3K/Akt signalling pathway through reversing PIP3 phosphorylation back to PIP2, leading to limited Akt activation, decreasing neuroinflammatory responses [4,152]. Recent research has unveiled new aspects of PTEN’s influence on the tumour microenvironment (TME), demonstrating its role in shaping immunosuppressive TME that impedes effective antitumour immune responses. PTEN deficiency within tumours can impair the activation of both the Type I IFN and NF-*κ*B pathways, fostering immunosuppression and tumour progression [153]. Additionally, the loss of PTEN triggers cellular senescence, acting as a protective mechanism against tumorigenesis [153]. PTEN’s influence extends to immune cell subsets, with PTEN deletion in mice revealing defects in T-cells, Treg cells, and B-cells. Notably, myeloid cell-specific PTEN deficiency augments phagocytic ability, reducing inflammation, and enhances resistance to infection [153]. PTEN dysfunction can occur from mutation or oxidative inhibition, leading to unregulated PI3K/Akt activation. These deleterious mechanisms of PTEN dysfunction can lead to the subsequent production of ROS owing to Akt activation and proinflammatory consequences [4,48,154]. With such limitations to the natural regulation of PI3K/Akt, the utilisation of inhibition mechanisms to manipulate the activity of this signalling cascade are advantageous.

Targeting the PI3K/Akt signalling pathway offers potential for modulating neuroinflammatory responses, as PI3K’s dual role in both promoting neuroprotection and driving harmful inflammation is context-dependent.

PI3K has been implicated in both proinflammatory and anti-inflammatory responses, but the exact factors governing the switch between these opposing functions remain unclear. Adding to the complexity, PI3K interacts with multiple signalling pathways, such as MAPK, STAT, mTOR, NF-*κ*B, GSK-3, Nrf2, Rho-GTPase, TGF-β1, and NLRP3 [155], leading to diverse and sometimes conflicting outcomes, making it challenging to predict the net effect of PI3K activation or inhibition in specific inflammatory settings.

Additionally, the effects of PI3K on inflammation may vary depending on the tissue type, the stage of inflammation, and the specific immune cell type involved. Thus, what may be beneficial in one context may be detrimental in another, leading to conflicting findings in different experimental models and disease conditions.

The timing and duration of PI3K activation has also been shown to be crucial in determining its proinflammatory or anti-inflammatory effect, emphasising the need for temporal control of PI3K signalling during neuroinflammation, in relation to TLR activation and downstream signalling [156]. Moreover, PI3K inhibitors may have pleiotropic effects, affecting multiple isoforms or having off-target effects, leading to nonspecific outcomes that complicate the interpretation of experimental results and clinical trials targeting PI3K in neuroinflammation.

### 5.1. PI3K Inhibitors

Various PI3K inhibitors have been explored in clinical and preclinical studies for their potential to modulate neuroinflammation by targeting specific PI3K isoforms. PI3K inhibitors can either block one or more isoforms, affecting different immune cells and pathways, depending on the target.

Among them, Wortmannin, a potent irreversible inhibitor for all Class I isoforms, has shown efficacy in reducing neuroinflammation and promoting neuronal survival but is limited in clinical use due to significant toxicity and off-target effects, such as lymphocytopenia and liver toxicity [157,158,159,160,161]. LY294002, a selective PI3K inhibitor derived from Quercetin by Lilly research laboratories, exhibits remarkable selectivity towards all Class I PI3K isoforms [158,162,163]. In experimental models, LY294002 has demonstrated potential in decreasing CNS inflammatory activation and the production of proinflammatory cytokines, indicating a neuroprotective nature. The translation of LY29004 into clinical applications has been limited due to adverse effects, such as dose-related and time-dependent issues [154,164,165].

AS605240, a selective PI3Kγ inhibitor, has shown potential in modulating immune cell activity and improving the outcomes in models of MS, lupus, and rheumatoid arthritis [29,166]. AS605240-induced PI3Kγ inhibition improved MS clinical scores, myelination, and axon numbers, correlating with reduced levels of CCL2 and CCL5 in the CNS [71]. However, AS605240 is 7.5 times more selective for PI3Kγ versus PI3Kα, which is unfavourable in preclinical outcomes due to the potential off-target inhibition of PI3Kα [71].

The pan-Class I PI3K inhibitor, ZSTK475, has shown a reduction in inflammation and disease progression in RA and EAE mouse models. A key challenge with pan inhibition is the concern over adverse effects from inhibiting PI3Kα and/or PI3Kβ alongside PI3Kδ and/or PI3Kγ [29]. CAL-101 (idelalisib), a selective PI3Kδ inhibitor, has shown remarkable results in follicular lymphoma and chronic lymphatic leukemia clinical trials, additionally, showing promise in modulating immune responses in preclinical models [167]. Idelalisib inhibition is able to regulate microglial phagocytosis [167]. Additionally, this inhibitor shows markedly diminished level of TNFα, IL-13, and IL-12 in human-monocyte-derived dendritic cells, presenting potential implications in regulating microglial activity [167]. Duvelisib (IPI-145), a dual inhibitor of PI3Kγ and PI3Kδ, is currently undergoing preclinical trials for inflammatory conditions [168]. Its dual targeting makes it a potential candidate for CNS diseases with neuroinflammatory components, as it impacts both microglia and T-cell activity [169,170].

These diverse PI3K inhibitors hold immense potential for deeper exploration in treating neuroinflammatory conditions and neurological disorders characterised by neuroinflammation. Their targeted actions within the intricate PI3K signalling network may unveil novel therapeutic strategies with far-reaching implications in the field.

### 5.2. Modulation of PI3K During Neuroinflammation

The modulation of PI3K activity offers significant therapeutic promise in managing neuroinflammation. Targeting specific PI3K isoforms in CNS immune cells, such as microglia and astrocytes, could reduce the detrimental effects of chronic inflammation, while preserving the protective aspects of the immune response. Isoform-specific inhibitors, such as AS605240 (PI3Kγ) and idelalisib (PI3Kδ), are especially promising for neuroinflammatory diseases due to their ability to selectively target immune cells involved in CNS pathology.

However, there has been limited research indicating that inhibition is applied to PI3K activity during neuroinflammation. Research has shown evidence of PI3Kδ and PI3Kγ isoforms being prominently expressed in immune cells, such as microglia and astrocytes [34,36]; thus, studies into these select isoforms may offer positive prospects surrounding the inhibitory effects of PI3K during neuroinflammation. Investigation into PI3Kγ inhibitors reducing neuroinflammation can be supported by a study indicating improvements in brain oedema and the neurological deficits of mice models after surgical brain trauma [171]. Inhibition exhibited relationships with reductions in immune cell infiltration, activation, and cytokine release, hence accounting for improvements in neuroprotection [171]. Targeting PI3K has presented promising neuroprotective elements: studies reveal decreases in microglial secreted TNF-⍺, with results showing increases in survival and prevention in the cognitive decline of AD mouse models [30].

PI3Kδ regulates inflammatory and oncological processes with implications on the release of TNF from macrophages and the release of cytokines from other immune cells. PI3Kδ expressed in microglia is necessary for the release of TNF-⍺ following glucose deprivation and restoration during ischemic stroke. It was found that mouse models subject to ischemia and reperfusion with applied selective PI3Kδ inhibition showed a reduction in neuronal damage and improved future outcomes [126]. PI3Kδ genetically and pharmacologically inactivated within mouse models, indicating the improvements of ischemia with a reduction in TNF levels, achieving protective results within the brain [126].

### 5.3. Trials for PI3K Modulation

While most clinical trials for PI3K inhibitors have focused on cancer, there has been growing interest in applying these inhibitors to neurodegenerative diseases characterised by neuroinflammation (Table 3). Several preclinical trials have explored the potential of PI3K inhibitors in various neurological conditions. In a mouse model of intracerebral haemorrhage (ICH), PI3Kγ inhibition demonstrated promising results by reducing the inflammatory response, neuronal damage, and brain dysfunction [172]. The specific inhibitor used in this study led to the decreased expression of proinflammatory genes in microglia and a subsequent reduction in neuronal death, ultimately improving neurological outcomes in animals [172,173].

In mouse models with 1-methyl-4-phenyl-1,2,3,6-tetrahydropyridin (MPTP)-induced PD, the PI3K inhibitor LY294002 exhibited neuroprotection by inhibiting inflammation and autophagy processes. This resulted in preserved dopaminergic neurons and improved motor function. Additionally, a pan-PI3K inhibitor, ZSTK474, was found to attenuate rheumatoid arthritis and experimental autoimmune encephalomyelitis neuroinflammation in animal models [174,175].

Another study explored the effect of PI3Kγ inhibition on tauopathy in a mouse model and found that the pharmacological inhibition of PI3Kγ reduced neuroinflammation and histopathological features associated with tauopathy, suggesting potential therapeutic benefits in tau-related diseases [176,177,178]. These preclinical trials provide valuable insights into the potential of PI3K inhibitors for the treatment of neuroinflammatory conditions and neurological diseases warranting further investigation in clinical settings.

Similarly, mouse models with induced early onset AD illustrate that the PI3Kγ inhibitor, AS605240, showed neuroprotective effects by attenuating neuroinflammation and enhancing long-term neurological function [179]. The inhibition of PI3K led to reduced microglial activation and proinflammatory cytokine production, improving cognitive function recovery in surgical brain injury models [171]. In ICV-STZ-induced AD mouse models, PI3Kγ inhibitors, such as AS605240, have demonstrated neuroprotective effects by reducing neuroinflammation and enhancing cognitive performance [180]. Early studies suggest that targeting PI3Kγ during neuronal injury could reduce amyloid-beta accumulation and neurotoxicity. Although, promising the transition from preclinical models to human trials has been slow. Currently, no PI3K inhibitors have been approved specifically for neuroinflammatory diseases, though several agents, such as duvelisib and copanlisib (for cancer), are in advanced clinical trials and could potentially be repurposed for CNS diseases [181].

There are no currently registered clinical trials specifically investigating PI3K inhibitors as standalone therapeutics for neuroinflammation or neurological diseases characterised by neuroinflammation processes. For instance, the NCT03372057 trial is a Phase II study evaluating the dose optimisation of duvelisib, a dual PI3Kγ and PI3Kδ inhibitor, in patients with relapsed or refractory peripheral T-cell lymphoma (PTCL) [182]. Another Phase II trial into NCT01882803 is investigating the safety and efficacy of duvelisib in patients with indolent non-Hodgkin lymphoma [183]. Additionally, the NCT02049515 trial in its third phase is evaluating the use of duvelisib in combination with ofatumumab for the treatment of chronic lymphocytic leukemia (CLL) or small lymphocytic lymphoma (SLL) [184]. While these trials provide valuable insight into the safety and efficacy of PI3K inhibitors, further research is needed to explore their potential use in the context of neuroinflammatory diseases.

Currently, there are no FDA-approved PI3K inhibitors specifically indicated for the treatment of neuroinflammatory conditions or neurological diseases characterised by neuroinflammation. However, several PI3K inhibitors have been approved for the treatment of certain types of blood cancers. Idelalisib (Zydelig) is a selective inhibitor of PI3Kδ and is FDA-approved for the treatment of relapsed or refractory CLL and relapsed follicular B-cell non-Hodgkin lymphoma [185,186,187]. Copanlisib (Aliqopa) is another approved PI3K inhibitor selectively targeting both PI3K⍺ and PI3Kδ. It is indicated for the treatment of relapsed B-cell non-Hodgkin lymphoma in patients who have received at least two prior systemic therapies [188,189]. Additionally, duvelisib (Copiktra), a dual inhibitor of PI3Kδ and PI3K⍺, is FDA-approved for the treatment of relapsed or refractory CLL and small lymphocytic lymphoma [75,190]. While these drugs have demonstrated efficacy in the treatment of specific blood cancers, their use in neuroinflammatory conditions and neurological diseases remains to be explored further.

A combination of preclinical modelling and clinical trials outline PI3K as an optimistic target for therapeutic manipulation. Proposing the isoform-specific inhibitors of Class I PI3Ks for therapeutic applicates toward breast cancer tumours with PI3K-dependence, melanoma, non-Hodgkin lymphoma, as well as additional cancer types [59,63,191,192,193]. This research has paved the way for PI3K inhibition as a therapeutic target, highlighting a greater understanding of the key mechanisms and prospects in generating new inhibitors to treat devastating disorders.

### 5.4. The Current Climate of PI3K Inhibitors

The current landscape for PI3K inhibitors is heavily focused on oncology, with several agents already approved for cancer treatment. However, the potential for these inhibitors to modulate neuroinflammation is gaining attention, particularly in diseases such as AD, PD, and MS, where chronic inflammation is a key driver of disease progression.

Investigation into selectivity interactions, mutagenesis, inhibitor design, and further structural analysis would be highly beneficial to fill gaps in the knowledge regarding PI3K inhibition. Additional obstacles in the identification of PI3K inhibitors are drug-related toxicity with examples of limited target inhibition, off-target binding, systemic PI3K inhibition, and the feedback upregulation of compensatory mechanisms [58,194]. The potential solutions of such limitations may be feasible using isoform-specific inhibitors rather than universal-PI3K or dual-PI3K inhibitors.

BBB permeability is one of the most significant challenges faced when developing PI3K inhibitors for CNS diseases. Many PI3K inhibitors, such as Wortmannin and LY294002, have poor BBB permeability, limiting their use for neurological conditions. Nanoparticle delivery systems and peptide shuttles offer promising strategies for the efficient transportation of PI3K inhibitors across the BBB. Lipid-based nanoparticles (liposomes, solid lipid nanoparticles) encapsulating PI3K inhibitors improve solubility and circulation time, whilst modifying the surface with ligands, such as transferrin or lactoferrin, enables receptor-mediated BBB penetration [195,196]. Similarly, exosome-based nanoparticles, which mimic natural vesicles, enter the brain through endocytosis with high biocompatibility and low immunogenicity [197]. Peptide shuttles further improve delivery, with receptor-targeting peptides, such as angiopep-2, binding to transport receptors, such as LRP1, to facilitate BBB penetration [198]. These delivery methods significantly enhance the use of PI3K inhibitors in the brain by overcoming the restrictive nature of the BBB, while minimising systemic toxicity.

Due to PI3K having several isoforms, isoform specificity is extremely important, and broad inhibitors can lead to nonspecific inhibition, causing off-target effects. Targeting specific isoforms such as PI3Kγ or PI3Kδ is crucial to avoid unwanted systemic immune suppression, while effectively modulating neuroinflammation. PI3K inhibitors could also disrupt normal immune function, leading to the increased risk of infections or immune dysregulation. Additionally, the long-term inhibition of PI3K pathways may impair processes such as neurogenesis and synaptic plasticity, leading to unintended consequences for brain health.

The translation of PI3K inhibitors for neuroinflammation faces several challenges that need to be addressed for successful therapeutic development. One major challenge is achieving isoform specificity, ensuring that PI3K inhibitors selectively target the isoforms involved in neuroinflammation, while avoiding off-target effects on other tissues and cell types. Implications in the selective targeting of immune cells involved in neuroinflammation is essential, as PI3K inhibitors should modulate the immune response, while preserving the functioning of other cell types, including microglia, astrocytes, neurons, and other immune cells, making it necessary to comprehensively understand the cellular crosstalk involved to avoid the unintended consequences of targeting PI3K in one cell type. Future studies should focus on understanding how PI3K signalling mediates this crosstalk and how modulating PI3K activity in one cell type can affect the behaviour of others.

Despite the progress in cancer therapies, the application of PI3K inhibitors to CNS diseases faces regulatory challenges due to the need for long-term safety data and the complexity of neuroinflammatory mechanisms. Achieving isoform specificity and ensuring BBB penetration are crucial for successful clinical translation. While preclinical studies show the short-term benefits of PI3K inhibition, the long-term effects of modulating this pathway—especially in chronic neurodegenerative diseases—remain unclear. Future research should prioritise understanding the safety profile of PI3K inhibitors over extended periods, as well as their potential impact on neurogenesis, synaptic plasticity, and overall CNS function.

The timing and duration of PI3K inhibitor application is critical during neuroinflammation modulation, as chronic inflammation may have different effects on the inflammatory response and neuroprotection. The complexity of neuroinflammatory processes, involving multiple signalling pathways and mediators, necessitates a multifaceted approach beyond targeting PI3K alone. Thus, a potential side effect of PI3K inhibition may induce adverse effects or interfere with normal cellular function in nontarget tissues.

There is currently limited research into the long-term effects of PI3K inhibition. However, it should be noted that the long-term inhibition of PI3K may have detrimental effects on the CNS by impairing synaptic plasticity, disrupting glial cell function, and increasing the risk of neurodegeneration. PI3K signalling is crucial in LTP and NMDA/AMPD receptor trafficking, essential for learning and memory; prolonged inhibition disrupts these processes, leading to cognitive decline. Sustained PI3K suppression in glial cells has been shown to impair the balance between proinflammatory and anti-inflammatory states. This disruption may induce excessive protective mechanisms that contribute to neuronal damage and worsening neuroinflammation. The long-term loss of PI3K-mediated regulation in glial cells weakens their ability to contain inflammation, clear cellular debris, and respond to metabolic stress, further impairing neuroprotective functions.

Despite promising preclinical data, the lack of clinical efficacy in translating PI3K inhibitors to human patients is a significant challenge due to differences in disease pathology and drug responses between animal models and humans.

Overcoming the challenge of BBB permeability remains a critical barrier to effective PI3K-targeted therapies in the CNS. Advances in brain-penetrant inhibitors that can selectively target PI3K isoforms in CNS immune cells, while minimising off-target effects, will be crucial for future therapies. Additionally, combination therapies targeting both PI3K and other pathways involved in neuroinflammation (e.g., mTOR, NF-κB) may offer a more comprehensive approach to managing neurodegenerative diseases.

Given the heterogeneous nature of neuroinflammatory diseases, future therapeutic strategies should consider personalised approaches. Tailoring PI3K-targeted therapies based on the specific neuroinflammatory profile of each disease could improve efficacy and minimise side effects. Biomarker identification and the development of companion diagnostics will be important for stratifying patients and optimising treatment.

## 6. Conclusions

Neuroinflammation is a hallmark of many neurodegenerative diseases, contributing significantly to their progression and global burden. The PI3K signalling pathway has emerged as a pivotal regulator of neuroinflammatory responses across multiple CNS cell types, including microglia, astrocytes, oligodendrocytes, and neurons. PI3K not only regulates immune cell activation and cytokine production but also modulates cell survival, differentiation, and tissue repair mechanisms. As such, targeting the PI3K pathway presents a promising therapeutic strategy for addressing chronic neuroinflammation in diseases such as AD, PD, and MS.

Throughout this review, we have discussed the involvement of specific PI3K isoforms in regulating immune responses in CNS cells. PI3Kγ and PI3Kδ isoforms play key roles in microglial and astrocytic activation during neuroinflammation, while PI3Kα and PI3Kβ contribute to neuronal survival and synaptic function. The dysregulation of PI3K signalling contributes to the chronic neuroinflammation and neuronal damage seen in neurodegenerative diseases. The selective inhibition of PI3K isoforms has shown potential in preclinical models, reducing neuroinflammation and promoting neuroprotection by modulating immune cell responses, protecting oligodendrocytes, and preserving neuronal function.

The therapeutic potential of PI3K inhibitors is considerable. Preclinical studies have demonstrated that selective PI3K inhibitors—such as AS605240 (PI3Kγ) and idelalisib (PI3Kδ)—can reduce neuroinflammatory responses, improve neurological outcomes, and protect against neurodegeneration. However, isoform specificity and BBB permeability remain critical challenges for translating these findings into clinical therapies for CNS diseases. The development of brain-penetrant PI3K inhibitors that specifically target dysfunctional immune cell activity without affecting systemic immune responses could significantly advance neuroinflammatory treatments.

Despite their promise, PI3K inhibitors must overcome challenges such as off-target effects, long-term safety concerns, and interference with normal immune functions. While some PI3K inhibitors have been approved for cancer treatment, their application to neuroinflammatory diseases remains largely unexplored in clinical settings. Nonetheless, the dual targeting of PI3K isoforms in microglia, astrocytes, and neurons offers an exciting opportunity to reduce neuroinflammation and prevent neurodegeneration in diseases that currently have limited treatment options.

Despite encouraging preclinical results, gaps in the current research need to be addressed to fully realise the potential of PI3K-targeted therapies for neuroinflammatory diseases. Key areas for future studies include isoform-specific targeting, crosstalk between CNS cells, BBB permeability, personalised medicine, and long-term safety and efficacy.

This review aimed to bring PI3K back into the spotlight and offer new insight into the complex role it plays in neuroinflammation. To further elucidate its mechanisms, future research should prioritize several key areas. Researchers should focus on the interactions between PI3K signalling pathways and other accessory molecules involved in the neuroinflammatory process. A deeper understanding of how these pathways overlap and work independently will offer more clarity on the specific contributions of PI3K to neuroinflammation. Additionally, attention should be given to exploring how the suppression of PI3K activity may be a better option to complete inhibition. Selective suppression will allow medical practitioners the opportunity to tune PI3K’s activity to best suit patient needs, thereby reducing off-target effects or unwanted physiological consequences. These targeted strategies will not only expand our knowledge of the PI3K pathway but also have direct implications for drug development. By refining our understanding of pathway interactions and selectively modulating PI3K activity, researchers will be better equipped to design tailored therapeutics that mitigate neuroinflammation, while minimizing the risk of adverse effects.

In conclusion, PI3K-targeted therapies hold significant promise for modulating neuroinflammation and offering new treatments for neurodegenerative diseases. However, translating this potential into clinical success requires overcoming several challenges. As research progresses, a more nuanced understanding of PI3K signalling in different CNS cell types and disease contexts will be critical for developing effective, personalised therapeutic strategies. By bridging the gaps in our current understanding, PI3K-targeted therapies could provide much-needed relief for patients suffering from the devastating effects of neuroinflammation and neurodegeneration.

## Figures and Tables

**Table 2:** Summary of PI3K isoform roles in CNS immune cells.

Cell Type	PI3K Isoforms	Effects on Neuroinflammation	Disease Involvement	Therapeutic Targeting
Microglia	PI3Kδ, PI3Kγ	Regulates activation and cytokine release (M1/M2)	MS, ALS	PI3Kδ/γ inhibitors to reduce inflammation
Astrocytes	PI3Kδ, PI3Kγ	Influences cytokine production (A1/A2) and glial scarring	AD, PD, MS	Isoform-specific inhibitors to reduce cytokine production and scarring
Oligodendrocytes	PI3Kγ, PI3Kα	Promotes survival and myelination	MS	PI3Kγ inhibitors to enhance remyelination
Neurons	PI3Kα, PI3Kγ	Regulates survival, synaptic plasticity	AD, PD, ALS	PI3Kα inhibitors to reduce synaptic loss and oxidative stress

**Table 3:** Summary of PI3K inhibitor trials.

Inhibitor	Target (PI3K Isoform)	Disease	Mechanism of Action	Clinical/Preclinical Trial Stage
AS605240	PI3Kγ	Inflammatory diseases (rheumatoid arthritis)	Selective inhibition of PI3Kγ, reducing leukocyte activation and migration.	Preclinical (in vitro and in vivo)
LY294002	Pan-PI3K	Cancer, neurodegeneration	Reversible inhibitor of PI3Kα, β, and γ; blocks cell proliferation and survival.	Preclinical (in vitro and in vivo)
ZSTK474	PI3Kα, β, δ	Solid tumours, glioblastoma	Suppresses tumour growth by inhibiting cell cycle progression through PI3K blockage.	Phase I
Duvelisib	PI3Kδ, γ	Chronic lymphocytic leukemia (CLL), lymphoma	Inhibits PI3Kδ in B-cells an PI3Kγ in T-cells, suppressing tumour microenvironment signalling.	FDA-approved for CLL (Phase III)
Copanlisib	PI3Kα, δ	Lymphoma, solid tumours	Inhibits PI3Kα and δ, blocking tumour proliferation and angiogenesis.	FDA-approved (Phase III)
NCT03372057	PI3Kδ	Marginal zone lymphoma	Clinical trials evaluating efficacy of PI3Kδ inhibitors in patients with relapsed/refractory lymphoma.	Phase II
NCT01882803	PI3K/mTOR	Lymphoma, relapsed CLL	Testing dual PI3K/mTOR inhibitors for safety and efficacy in haematological cancers.	Phase I
NCT02049515	PI3Kδ	Follicular lymphoma	Examines long-term safety and efficacy of PI3Kδ inhibitors.	Phase II
Ofatumumab	Anti-CD20 (indirectly linked to PI3K pathway)	Chronic lymphocytic leukemia	Anti-CD20 monoclonal antibody depletes B-cells, indirectly modulating PI3Kδ signalling.	FDA-approved (Phase III)
Idelalisib	PI3Kδ	CLL, non-Hodgkin’s lymphoma	Selectively inhibits PI3Kδ, reducing B-cell receptor signalling and proliferation.	FDA-approved (Phase III)
